# Frosted branch angiitis due to cytomegalovirus-associated unmasking immune reconstitution inflammatory syndrome: a case report and literature review

**DOI:** 10.1186/s12879-021-06311-4

**Published:** 2021-06-26

**Authors:** Shi Tang, Ning Zhao, Li Yang Wang, Ying Wen

**Affiliations:** 1 Infectious Diseases Department, The First Affiliated Hospital of China Medical University, No. 155, Nanjing North Street, Heping District, Shenyang, 110001 Liaoning Province China; 2 Department of Ophthalmology, The First Affiliated Hospital of China Medical University, Shenyang, Liaoning Province China; 3 Department of Gastroenterology, The sixth People’s Hospital of Shenyang, Shenyang, Liaoning Province China

**Keywords:** Frosted branch angiitis, Anti-cytomegalovirus treatment, Unmasking immune reconstitution inflammatory syndrome, Case report

## Abstract

**Background:**

Cytomegalovirus (CMV) retinitis is a common opportunistic infection in patients with acquired immunodeficiency syndrome. The common funduscopic manifestations are haemorrhagic necrotising variety and granular variety. Frosted branch angiitis (FBA), as a special form, when it occurred after antiretroviral therapy(ART), could possibly be associated with immune reconstitution. We report a case of FBA secondary to CMV infection-associated unmasking immune reconstitution inflammatory syndrome (IRIS).

**Case presentation:**

A 27-year-old man with human immunodeficiency virus infection developed FBA after 35 days of ART. The left Aqueous humour (AqH) tested positive for CMV DNA, and the patient was diagnosed with CMV retinitis. The degree of intraocular inflammation was reflected by increased levels of interleukin (IL)-6 and IL-8 in AqH. After anti-CMV treatment and continuous ART for several months, his FBA and vision significantly improved. CMV DNA became undetectable in the left AqH, and the IL-6 and IL-8 levels in AqH decreased.

**Conclusion:**

FBA could be a sign of CMV-associated unmasking IRIS. Anti-CMV treatment alone or combination with steroid treatment may be administered, depending on the changes in CMV DNA load and immunologic profile of AqH.

## Background

Frosted branch angiitis (FBA) is a special form of vasculitis, affecting the entire retina. The funduscopic findings of FBA include bilateral widespread retinal vasculitis with severe sheathing of the retinal vessels, resembling frosted branches of a tree, especially at the periphery, and mild to moderate iritis or vitritis. In this article, we reported a case of FBA secondary to cytomegalovirus (CMV) infection-associated unmasking immune reconstitution inflammatory syndrome (IRIS).

## Case presentation

**Fig. 1:**
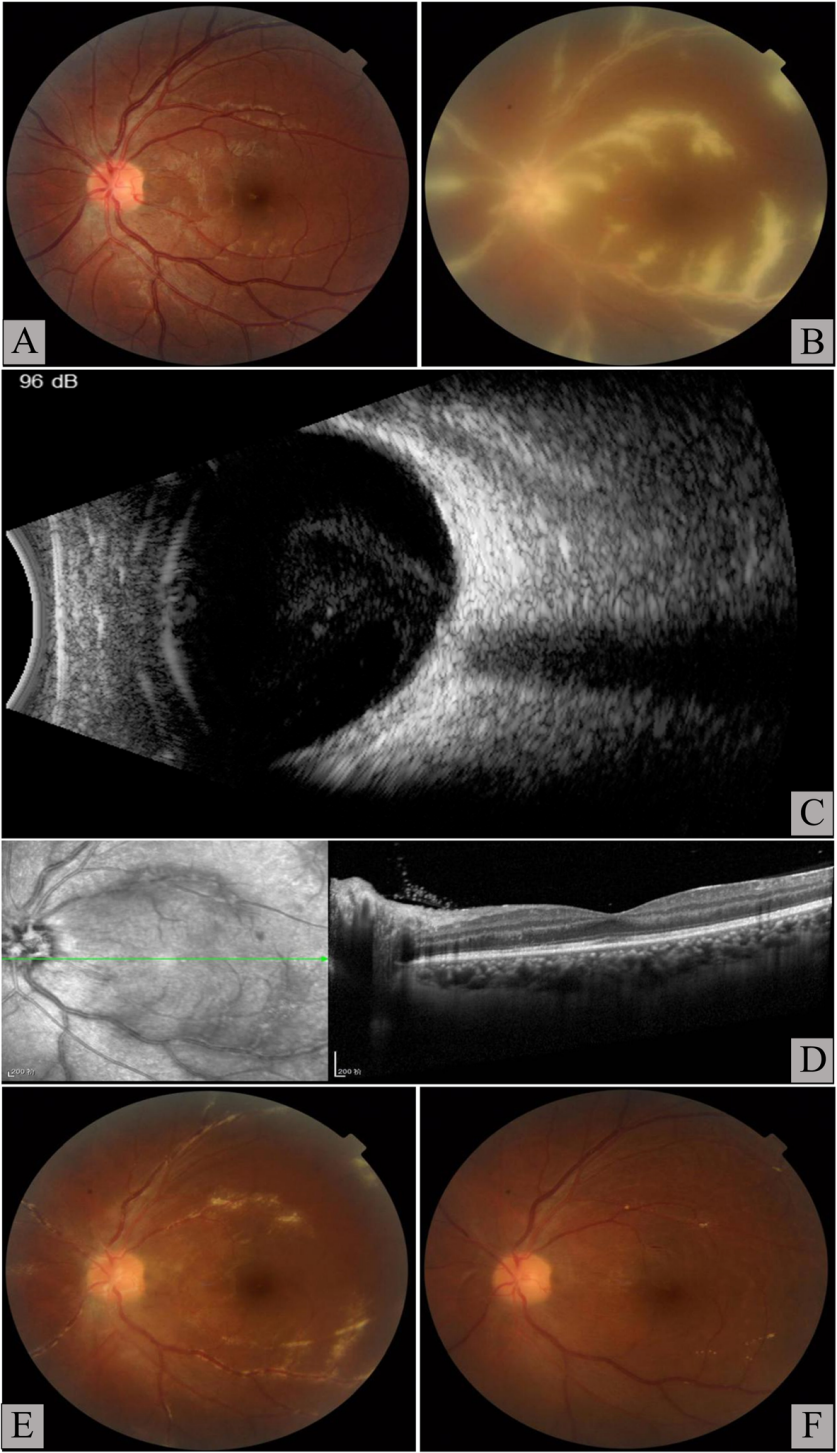
Changes observed in the patient’s eye examination. **A** Normal bilateral eyes appearance before antiretroviral therapy (ART). **B** After 5 weeks of ART, the left eye showed sheathing of the retinal vessels appearing like frosted branches of a tree without haemorrhages, necrosis, and occlusion. **C** Eye ultrasound revealed mild vitreous haze in the left eye after 5 weeks of ART. **D** No oedema in the macular area by optical coherence tomography were seen after 5 weeks of ART. **E** After 6 weeks of anti-CMV treatment, frosted branch angiitis in the left eye had significantly improved. **F** After 6 months of anti-CMV treatment, the retinal perivenous exudate in the left eye had resolved

A 27-year-old young man, who previously had sexual contact with other men, was diagnosed with human immunodeficiency virus(HIV) infection 2 months ago. His CD4 + T cell count was 33 cells/μL. His serum anti-cytomegalovirus immunoglobulin M (IgM) was 23.6 U/mL (normal range: 0–18 U/mL), and CMV IgG was 139.0 U/mL (normal range:0–12 U/mL). His serum CMV DNA load was 4.54 × 10^3^ copies/mL. The patient had no ocular symptoms and signs. No abnormalities were found on funduscopic screening examination (Fig. 1A). The preemptive anti-CMV treatment was not performed. His acid-fast smear of sputum, interferon-gamma release assay for *Mycobacterium tuberculosis*, and tuberculin skin test were negative. The IgM antibodies of rubella virus, herpes simplex virus(HSV), varicella-zoster virus(VZV), and Epstein-Barr virus(EBV) were all negative. The IgM and IgG antibodies of toxoplasma gondii, mycoplasma, and legionnella were all negative. The specific antibody of syphilis was negative. The blood sugar level was normal. Abdominal ultrasound screening, chest computed tomography, and brain magnetic resonance imaging were normal. Then, the patient received antiretroviral therapy (ART), including tenofovir, lamivudine, and efavirenz as well as oral co-trimoxazole, two tablets daily, for primary prophylaxis of *Pneumocystis jirovecii* pneumonia.

On the 35th day of ART, the patient complained of floaters and blurred vision in the left eye and was admitted to our hospital due to worsening of eyesight. No other systemic abnormal symptoms and signs had been found, such as fever, rashes, cough, watering nose, sneeze, sore throat, parotid enlargement, mouth ulcer, diarrhoea, and bloody stool, etc. His CD4 + T cell count rapidly increased to 172 cells/μL. The HIV RNA load was 1.80 × 10^3^ copies/mL. The left and right eyes were not red and swollen. His visual acuity was 20/20 in the right eye and 20/30 in the left eye. Bilateral intraocular pressures were normal. Slit-lamp examination showed diffuse punctate keratic precipitates and positive aqueous flare in the left eye. The size of the pupils and the light response were normal. The lens was transparent. Fundal examination revealed extensive retinal perivenous exudate, forming frosted branch vascular sheathing in the left eye (Fig. 1B). At the lower border of the right eye, peripheral retinitis, characterized by white granular exudates with minimal haemorrhage, was detected. Eye ultrasound revealed a slightly vitreous haze (Fig. 1C). Optical coherence tomography did not reveal macular oedema (Fig. 1D). The serum CMV DNA became negative (< 1.0 × 10^3^ copies/mL). The level of C-reactive protein was 5.60 mg/L (normal range:0.00–5.00 mg/L). The levels of antistreptolysin O and rheumatoid factor were normal. Blood screening results of autoantibodies were all negative. A 100 μL sample of Aqueous humour (AqH), collected by anterior chamber puncture, was sent to Beijing Zhi De medical laboratory science finite company. CMV DNA in the left AqH was 6.54 × 10^5^copies/mL. Using BD-Pharmingen cytometric bead array, the interleukin (IL)-6 and IL-8 levels in the AqH were 2845.0 pg/mL (normal range: 1–50 pg/mL) and 967.8 pg/mL (normal range: 0–20 pg/mL), respectively. The DNA of HSV, EBV, and VZV were all negative in the left AqH. He was treated with intravitreal ganciclovir (2 mg twice a week for a total of four times) and intravenous ganciclovir (5 mg/kg twice daily for 2 weeks, followed by 5 mg/kg/day for 1 month). No non-steroidal anti-inflammatory drug was prescribed for the patient. His previous ART regimen was continued. Oral co-trimoxazole, two tablets daily, was continued. His FBA significantly improved (Fig. 1E). Thereafter, he had to switch to foscarnet at 180 mg/day because of leukopenia. Subsequently, foscarnet was replaced with oral ganciclovir (3 g/day). During the sixth-month follow-up, the peripheral granular lesion in his right eye subsided, and the vascular sheath-like exudates in the left eye resolved (Fig. 1F). His visual acuity became 20/25 in the left eye. His serum CMV DNA was undetectable. CMV DNA in the left AqH was also negative (< 1.0 × 10^3^ copies/mL), and the levels of IL-6 and IL-8 in the left AqH decreased to 28.5 pg/mL and 5.6 pg/mL, respectively. His CD4 + T cell count was 131 cells/μL, and HIV RNA load was 30.3 copies/mL. His serum CMV IgM was negative, and CMV IgG exceeded 180.0 U/mL.

## Discussion and conclusion

This was the first case that met the criteria for diagnosing early unmasking IRIS-FBA [1]. His condition was classified as a re-activation of a latent CMV infection. Short-term ART decreased the CMV DNA in his blood to undetectable levels [2]. However, subacute visual loss and floaters developed in his left eye. Detectable CMV DNA in the intraocular fluid is crucial for CMV retinitis diagnosis and differentiation from primary FBA or other infections. The elevated levels of IL-8 and IL-6 (principal cellular sources from monocytes and macrophages) in the AqH, as an indicator of active retinitis, obviously decreased following the anti-CMV treatment [3–5]. In this case, anti-CMV treatment monotherapy decreased the CMV DNA load as well as IL-8 and IL-6 levels in the AqH. Along with this, the patient’s eyesight improved, and perivascular exudation regressed. Thus, systemic steroid treatment was not required.

CMV retinitis is an important cause of blindness in individuals with advanced HIV infection and is characterized by intraretinal haemorrhages, white zones of retinitis, retinal oedema, and vasculitis. FBA is a special form of retinal vasculitis. This may be primary FBA or secondary to ocular infectious diseases, such as CMV, syphilis, HSV, VZV, tuberculosis, toxoplasmosis, and non-infectious diseases, such as autoimmune diseases and haematological malignancies in the non-HIV-infected population [6]. Antigen-antibody complex deposition and direct CMV infection of the vessel wall are the underlying pathogeneses [7, 8]. In the setting of HIV infection, FBA is an uncommon sign. Apart from the syphilis-related case [9], reported cases were exclusively associated with CMV retinitis [7, 8, 10–16] (summarised in Table 1). Currently, only two cases of FBA were associated with paradoxical IRIS [10, 12]. The time from ART initiation to IRIS development was 7 days [12] and 6 months [10], respectively. Anti-CMV treatment without steroid treatment was beneficial in ART-naive HIV-infected patients [7, 16]. However, some individuals responded well to steroid treatment, especially in the presence of CMV-associated IRIS [12, 13].

**Table 1:** Summary of reported cases with HIV-infection and cytomegalovirus-associated frosted branch angiitis

Case	Age (years)	Gender	CD4 cell count(/μl)	Eyes with FBA	Duration post-ART	ART	Outcome	Treatment
Mansour AM et al. 1993 [7]	27	M	NM	Left	NM	NM	R	Gancyclovir
	39	M	NM	Right	NM	NM	R	Gancyclovir
	24	M	NM	Left	NM	NM	NM	Gancyclovir
	35	M	NM	Left	NM	NM	R	Gancyclovir
	35	M	NM	Both	NM	NM	R	Introvenous gancyclovir
	32	M	NM	Both	NM	NM	R	Gancyclovir
R F Spaide et al. 1992 [8]	36	M	10	Both	NM	NM	R	Introvenous gancyclovir
	50	M	10	Both	NM	NM	R + retinal detachment	Gancyclovir, vitrectomy,intravenous foscarnet
	28	M	20	Right	NM	NM	R	Intravenous foscarnet
Mehmet Numan Alp et al. 2010 [10]	36	F	From 9 to 20	Both	6 m	Y NDR	R +retinal detachment	Introvenous gancyclovir, Periocular and topical steroids, ART continuation
Aguilar Lozano et al. 2016 [11]	41	M	31	Left	8 m	Y DR	NM	Introvenous gancyclovir, ART adjustment
Supinda Leeam-ornsiri et al. 2013 [12]	40	F	From 53 to 107	Right	1 W	Y NDR	R	Intravitreal ganciclovir injections, Oral prednisone ART continuation
H F Fine et al. 2001 [13]	7	M	30	Both	NM	NM	R	Introvenous gancyclovir and foscarnet, Oral prednisone
Biswas et al. 1999 [14]	39	M	69	Both	5 m	Y DR	R +retinal detachment	Introvenous gancyclovir, Vitrectomy, Intravitreal injections of gancyclovir
S A Geier et al. 1992 [15]	49	M	NM	Right	NM	NM	R	Introvenous gancyclovir, Oral fluocortolone
Feifei Mao et al. 2016 [16]	26	M	11	Right	3 W	Y NDR	R	Oral prednisone,intravitreal foscarnet injections, ART continuation
Our patient	27	M	From 33 to 172	Left	5 W	Y NDR	R	Intravitreal ganciclovir injection, Introvenous gancyclovir,intravenous foscarnet ART continuation

*M* male, *F* female, *NM* not mentioned or not done, *DR* drug resistance of ART, *NDR* non-drug resistance of ART, *R* regression of FBA, *Y* yes, *FBA* frosted branch angiitis, *ART* Antiretroviral therapy

In conclusion, FBA could be a sign of CMV-associated unmasking IRIS. In HIV-infected patients with asymptomatic CMV viremia, preemptive anti-CMV therapy was not recommended by the guideline. However, some trials assessing preemptive anti-CMV therapy in advanced HIV-infected patients documented its efficacy [17, 18]. CMV retinitis can be prevented by taking early ART and maintaining a CD4 + T cell count > 100 cells/mm^3^. Recognising the early manifestations of the disease and initiating proper therapy are crucial. Anti-CMV treatment with or without steroid treatment can be administered for FBA depending on the changes in CMV DNA load and immunologic profile of the AqH. In patients with no response to anti-CMV medications, systemic corticosteroids are recommended.

## Data Availability

Not applicable (no datasets were generated or analyzed during the current study).

## Abbreviations

AqH: Aqueous humour
ART: Antiretroviral therapy
CMV: Cytomegalovirus
EBV: Epstein-Barr virus
FBA: Frosted branch angiitis
HIV: Human immunodeficiency virus
HSV: Herpes simplex virus
Ig: Immunoglobulin
IL: Interleukin
IRIS: Immune reconstitution inflammatory syndrome
VZV: Varicella-zoster virus
